# Measurement of Trace Elements (Zinc, Copper, Magnesium, and Iron) in the Saliva of Horses: Validation Data and Changes in Equine Gastric Ulcer Syndrome (EGUS)

**DOI:** 10.3390/ani14121724

**Published:** 2024-06-07

**Authors:** Alberto Muñoz-Prieto, José Joaquín Cerón, Fernando Tecles, María Martín Cuervo, Maria Dolores Contreras-Aguilar, Ignacio Ayala, Adrián Oudada-Guillén, Luis Pardo-Marín, Sanni Hansen

**Affiliations:** 1 Interdisciplinary Laboratory of Clinical Analysis (Interlab-UMU), Regional Campus of International Excellence ‘Campus Mare Nostrum’, University of Murcia, Campus de Espinardo, 30100 Murcia, Spain; jjceron@um.es (J.J.C.); ftecles@um.es (F.T.); mariadolores.contreras@um.es (M.D.C.-A.); iayape@um.es (I.A.); adrian.oudada@um.es (A.O.-G.); lpm1@um.es (L.P.-M.); 2 Department of Animal Medicine, Faculty of Veterinary, University of Extremadura, Avda de la Universidad s/n, 10003 Cáceres, Spain; mariamc@unex.es; 3 Section Medicine and Surgery, Department of Veterinary Clinical Sciences, University of Copenhagen, Agrovej 8, 2630 Taastrup, Denmark; sannih@sund.ku.dk

**Keywords:** trace elements, horses, saliva, EGUS

## Abstract

**Simple Summary:**

This study aimed to assess the potential of spectrophotometric assays to measure trace elements (Zinc, Copper, Magnesium, and Iron) in horse saliva and their possible changes in Equine Gastric Ulcer Syndrome (EGUS). EGUS is prevalent in horses, influenced by intensive management conditions, and encompasses equine squamous gastric disease (ESGD) and equine glandular gastric disease (EGGD). Validated spectrophotometric assays were used to measure these elements in horses with ESGD, EGGD, both, and healthy horses. Results showed significantly lower Zinc and Magnesium levels in horses with EGGD, while Iron concentrations were lower in ESGD and EGGD cases. These findings suggest potential biomarkers for EGUS, shedding light on its pathophysiology and aiding in diagnosis and management strategies for improved equine health.

**Abstract:**

The objective of this study was to evaluate the possible use of spectrophotometric assays for the measurement of trace elements, including Zinc (Zn), Copper (Cu), Magnesium (Mg), and iron (Fe) in the saliva of horses and study their possible changes in equine gastric ulcer syndrome (EGUS). EGUS is a highly prevalent disease, with a current high incidence due to the increase in intensive management conditions. There are two EGUS diseases: equine squamous gastric disease (ESGD) and equine glandular gastric disease (EGGD), which can appear individually or together. For this purpose, automated spectrophotometric assays for measuring these analytes in horse saliva were analytically validated. Then, these analytes were measured in the saliva of horses with only ESGD, only EGGD, both ESGD and EGGD and a group of healthy horses. The methods used to measure the analytes were precise and accurate. Horses diagnosed with EGGD presented significantly lower levels of Zn and Mg. Fe concentrations were significantly lower in the saliva of horses with ESGD and EGGD. Overall, these results indicate that there are changes in trace elements in saliva in EGUS that could reflect the physiopathological mechanisms involved in this process and open the possibility of using trace elements as biomarkers of this syndrome.

## 1. Introduction

Equine gastric ulcer syndrome (EGUS) is a highly prevalent disease, with a current high incidence due to the increase in intensive management conditions, and it is also associated with increasing expectations in the performance of horses [1]. Two different diseases in EGUS have been described: equine squamous gastric disease (ESGD) and equine glandular gastric disease (EGGD), which can appear individually or together. Both diseases have different physiopathological mechanisms and etiology. In ESGD, the squamous mucosa of the gastric wall is damaged. This occurs due to increased acid exposure to this mucosa, and some predisposing factors have been identified, such as high-concentrate diets, diets with high starch content, and fasting for more than six hours. On the other hand, the exact mechanisms behind EGGD remain unclear, but it is believed to stem from a failure in the glandular mucosa’s normal defense systems [2]. The glandular mucosa is designed to endure constant exposure to gastric acid. Prostaglandins play a crucial role in safeguarding this lining by regulating blood flow, mucus and bicarbonate secretion, and acid production [3]. However, when these defense mechanisms fail, potentially due to disruptions in the hydrophobic surface of the mucosa, alterations in the bacterial microbiome, or stress, EGGD can develop [4]. In particular, stress has been shown to significantly impact these defenses, as horses with EGGD exhibit heightened adrenocortical responses to adrenocorticotropic hormone and new stimuli, indicating a strong connection between stress and the degradation of mucosal protection [4,5].

Trace elements such as Zinc (Zn), Copper (Cu), Magnesium (Mg), and Iron (Fe) have been related to gastric ulcers in humans and animals. In the case of Zn, the protective action of Zn ions on the gastric mucosa has been described [6,7]. In this line, Zn chloride pretreatment significantly reduced the volume of gastric secretion and the total acid output, as well as the incidence of gastric ulcers in rats induced either by stress or by acid accumulation [8]. In addition to the preventive effect, Zn can be used to treat gastric ulcers in humans [9]. In another study, daily administration of Zn sulfate reduced peptic ulcer size, and patients with normal serum zinc levels had better healing processes [10]. On the other hand, Zn deficiency has been associated with gastric ulcers in rats. This is likely because a decrease in Zinc produces an increase in total acid output, leading to a breakdown of the gastric mucosal barrier and an increase in gastric lesions [11].

Regarding Cu, Cu nicotinate and Cu gluconate have been described as having a protective effect and anti-ulcerogenic activity in rats, which is linked to a decrease in the production of thromboxane A_2_ (TXA_2_) levels [12]. In this line, Cu offers protection from several non-steroidal acidic anti-inflammatory drugs that cause ulceration, such as clopirac, niflumic acid, and aspirin. This protection is related to an inhibition of prostaglandin synthesis [13]. In addition to the inhibition of prostaglandin and thromboxanes, it has been postulated that Cu can effectively scavenge superoxide radicals and produce oxidative damage. It was found that in humans, the average Cu levels in patients’ serums with peptic ulcer disease are considerably higher than those in healthy ones, probably in order to produce protection against the disease [14].

Mg is known to be an important constituent of antacids, and it is used in humans for constipation and dyspepsia treatment. In rats, it is described to be anti-ulcerogenic due to its ability to decrease the number of parietal cells and increase the number of mucous cells in the stomach [15]. In the same study [15], authors found a significant reduction in the ulcer scoring of magnesium-pretreated ulcerated rats compared with ulcerated untreated groups. In addition, it has been reported that Mg can protect the stomach due to its ability to decrease the amount of acid secretion by decreasing the function of the parietal cells due to the inhibition of the calcium-signaling mechanisms involved in it [16]. Overall, Mg reduces both the number and function of parietal cells in the stomach. In addition, the administration of oral Mg reduces gastric mucosa susceptibility to injury in experimental diabetes mellitus. This could be related to the effect that Mg has in the reduction of serum nitric oxide and lipid peroxidation and the increase in gastric mucous cells [17]. In this line, gastric ulcers in mice by producing stress due to immobilization were significantly larger in mice with a low magnesium concentration in serum [18].

Fe can produce gastric ulcers when it is in high concentrations in the body. Ferrous (Fe^2+^) forms are normally taken up into duodenal enterocytes by the energy-dependent divalent metal transporter-1 carrier protein, but in high concentrations, they are also absorbed in a concentration-dependent fashion, leading to an increase in gastric intracellular ferric and ferrous ions [19]. These forms of Fe catalyze the production of reactive oxygen metabolites and lipid peroxidation and can lead to necrosis and inflammation of the stomach mucosae. These alterations, combined with the local ischemia that can be produced due to Fe-induced submucosal vascular thrombosis, can be involved in ulcer production [20]. In this line, in humans, Fe supplements, at overdose or even at therapeutic ranges, can produce injury in the mucosa of the upper gastrointestinal tract [19].

Saliva is used as a sample for the measurement of biomarkers that can be related to stress, the immune system, inflammation, redox status, and the general metabolism of individuals. In horses, it can be obtained by easy collection procedures without producing stress or pain, and in cases of EGUS, changes have been described in various biomarkers, showing even differences depending on the type of EGUS [21,22]. However, to the authors’ knowledge, there are no reports about trace elements in the saliva of horses.

The objective of this report was to evaluate the possible use of spectrophotometric assays for the measurement of the trace elements Zn, Cu, Mg, and Fe and study the possible changes in these analytes in the saliva of horses with EGUS. For this purpose, an analytical validation of automated spectrophotometric assays was performed to measure these analytes in horse saliva. Then, after the validation, these analytes were measured in the saliva of horses with ESGD, EGGD, both ESGD and EGGD and a group of healthy horses for comparative purposes.

## 2. Materials and Methods

### 2.1. Animals

This study included saliva samples from horses examined at the Large Animal Teaching Hospital at the University of Copenhagen and the Veterinary Teaching Hospital of the University of Extremadura. It covered the period from August 2020 to August 2023.

A thorough clinical assessment was conducted on all horses, encompassing a detailed medical history, a physical examination (which encompassed weight assessment, body condition score (BCS) measured on a nine-point scale [23], evaluation of heart and respiratory rates, rectal temperature, color of mucous membranes, capillary refill time, and borborygmus), along with hematological and biochemical analyses. Following a 16 h fasting period, gastroscopy was performed on all subjects in accordance with previously outlined protocols [24]. For the identification of EGSD and EGGD, the images obtained with gastroscopy were interpreted by only one person from each hospital, both with more than ten years of experience, according to the guidelines by the European College of Equine Internal Medicine (ECEIM) indicated in its Consensus Statement [23,24]. A horse was considered to have ESGD if it had a score higher than 1 when evaluated with the grading scale of ESGD created by the ECEIM Consensus, which goes from 0 to 4 points. A horse was considered to have EGGD if it had a score higher than 1 on a grading scale that goes from 0 to 4 as previously described [21]. Other complementary diagnostic procedures, such as a rectum exam, image exploration of the abdomen, centesis of the abdomen, or exploratory laparotomy, were performed if needed.

Based on the clinical evaluation and gastroscopy, horses were classified into the following two groups:

- EGUS group: Horses included in this group showed different clinical signs such as loss of weight, problems with riding, changes in behavior, and/or signs of pain, and if they had images compatible with EGUS at gastroscopy [25]. This group was comprised of horses diagnosed with EGUS and not having any other disease. Three different subgroups were performed in the EGUS group. One includes horses with only ESGD, another includes horses with only EGGD, and the third one has horses with ESGD and EGGD at the same time.
- Healthy horses: This group includes horses admitted for castration or other routine health examinations. All the horses included had no remarks on a clinical examination, hematological and biochemical results that remained within the reference values, and no ulcers detected at the gastroscopic examination.

### 2.2. Saliva Collection

Saliva samples were collected from all horses prior to intravenous sedation for gastroscopy. Saliva was collected using a synthetic sponge, which was then placed into a saliva collection tube (Salivette, Sarstedt, Aktiengesellschaft & Co., Nümbrecht, Germany), as previously reported [26]. For saliva collection, each animal was permitted to gently chew on a sponge (measuring 5 cm long and 2.5 cm wide) attached to a thin, flexible metal rod for approximately 1–2 min, ensuring thorough moistening of the sponge. Subsequently, each moistened sponge was placed into a Salivette tube and promptly stored on ice. Within 20 min of collection, the samples were transported to the laboratory. Upon arrival, the sponges were centrifuged at 3000× *g* for 10 min to extract saliva, which was then transferred to 1.5 mL tubes (Eppendorf Ibérica, Madrid, Spain) and preserved at −80 °C until analysis. The saliva samples included in this report did not have evident signs of dirtiness or contamination.

### 2.3. Zinc, Copper, Magnesium, and Iron Assays

Zn was measured using a colorimetric assay (Zinc, ZN2341, Randox Laboratories Ltd., Crumlin, UK). Cu was measured with a colorimetric method (Copper, CU2340, Randox Laboratories Ltd., UK). The principle of Zn and Cu methods was based on the chromogen 4-(3,5-dibromo-2-pyridylazo-N-Ethyl-N-(3-sulphopropyl) aniline (3,5-di-Br-PAESA) for Cu and the chromogen 2-(5-bromo-2-pyridylazo)-5-(N-propyl-N-sulphopropylamino)-phenol (5-Br-PAPS) for Zn [27]. Mg was determined using a colorimetric assay (11797, Magnesium, BioSystem S.A., Barcelona, Spain). In this method, the Mg of the sample reacts with xylidyl blue in an alkaline medium, forming a colored complex that can be measured by spectrophotometry. Fe was analyzed using a colorimetric method (Iron-Ferrozine, 11,509, BioSystems S.A., Barcelona, Spain) in which the ferric ion present in the sample and bound to transferrin is released by the action of guanidinium and reduced to ferrous forms by hydroxylamine. Then, the ferrous ion forms a colored complex with ferrozine that is quantified by spectrophotometry.

Measurements were made on automated chemistry analyzers (Olympus AU 400 for Zn, Cu, and Mg, and Olympus AU 600 for Fe, Olympus Diagnostica GmbH1, Hamburg, Germany).

### 2.4. Analytical Validation of Assays in Horse Saliva

In order to evaluate the precision of the assays, intra- and inter-assay imprecision was calculated. For this purpose, three samples of saliva that had different concentrations of the analytes obtained for different horses were used. The imprecision intra-assay was obtained by the analysis of each sample in a single analytical run five times. The imprecision inter-assay was obtained by the analysis of each sample in five runs that were made on five different days. To obtain the level of imprecision, the coefficient of variation (CV) was calculated by the formula SD/M and reported in percentage, being the SD the standard deviation of the measurements of the replicates and M the mean values of the replicates.

Accuracy was estimated indirectly by linearity under dilution, and in the case of Fe, since interference was detected with serial dilutions, it was performed with recovery experiments. The linearity under dilution was evaluated by serial dilutions of a saliva sample with a high concentration for each different analyte. The dilutions were made using ultrapure water. For each serial dilution, linear regression equations and determination coefficients (r2) were calculated between the observed values and the theoretically expected. In addition, a graphic representation was made. In the case of the recovery test, a sample with a low concentration was mixed with a sample with a high concentration of the analyte in the following proportions: 50–50%, 25–75%, and 75–25%. To calculate the percentage of recovery, the observed results were divided by the expected results, and the value of this division was multiplied by 100.

In addition, accuracy was evaluated by the comparison of the spectrophotometric assays with ICP-MS (Agilent 7900, Inductively Coupled Plasma–Mass Spectrometry, Santa Clara, CA, USA). This is a highly sensitive and accurate technique [28], validated for measuring trace elements in saliva, and is considered the gold-standard method for the measurement of Zn, Cu, Mg, and Fe [29]. Twenty-two saliva samples obtained from horses at the Large Animal Teaching Hospital at the University of Copenhagen were analyzed using an automated assay and the ICP-MS method. For the ICP-MS analysis, the samples were treated with acid HNO3 65% and analyzed with the ICP-MS described above.

The sensitivity of each assay was calculated by the detection limit (LOD). The LOD is defined as the lowest concentration of the analyte that can be differentiated from the values obtained with a zero-value sample. In our report, the detection limit was established by measuring a zero standard (ultrapure water) 20 times in the same analytical series and applying the following calculation: the mean of the 20 determinations plus 3×SD [30].

### 2.5. Zinc, Copper, Magnesium, and Iron in the Saliva of Horses with Equine Gastric Ulcer Syndrome

Concentrations of Zn, Cu, Mg, and Fe were measured and compared in the saliva of horses included in this study, as described at point 2.1.

### 2.6. Statistical Analysis

The data had a non-parametric distribution when they were evaluated for normality by the Kolmogorov–Smirnov test. Based on this, non-parametric tests were used for further statistical analysis. To evaluate the possible differences in trace elements between all horses with both types of EGUS, horses with EGGD, horses with ESGD, and healthy horses, a Kruskal–Wallis test, followed by Dunn’s test for multiple comparisons, was performed. The results were indicated as the median of the values and interquartile range (IQR) of each group. For the comparison of the results of the spectrophotometric assays and ICP-MS in the 22 saliva samples analyzed for Zn, Cu, Mg, and Fe, the Spearman correlation was performed. In all cases, a value of p lower than 0.05 was significant. All the calculations were made by the use of statistical software (GraphPad Prism 8, GraphPad Software, San Diego, CA, USA).

## 3. Results

### 3.1. Population of Horses Included

In this study, 80 horses were examined. Among them, the healthy group encompassed 16 animals (10 mares, 6 geldings) of various breeds. Their median age stood at 15.6 years (range: 6–23), with a Body Condition Score (BCS) averaging 5.9 (range: 4–8). Conversely, the EGUS group comprised 64 horses (30 mares, 34 geldings) representing diverse breeds. These horses had a median age of 11.6 years (range: 4–24) and a BCS of 5.2 (range: 4–8). Notably, within this group, 23 horses suffered from ESGD, 20 from EGGD, and 21 from both ESGD + EGGD. It is noteworthy that there were no statistically significant differences found when comparing the age and BCS among the groups.

### 3.2. Analytical Validation of the Assays

The Zinc assay showed mean intra- and inter-assay CVs of 5.76% and 6.27%. For the Cu, the mean intra- and inter-assay CVs were 9.45% and 12.30%. In the case of Mg, mean intra- and inter-assay CVs showed 7.22% and 7.59%. The mean intra- and inter-assay CVs for Fe were 8.52% and 9.54%.

**Figure 1:**
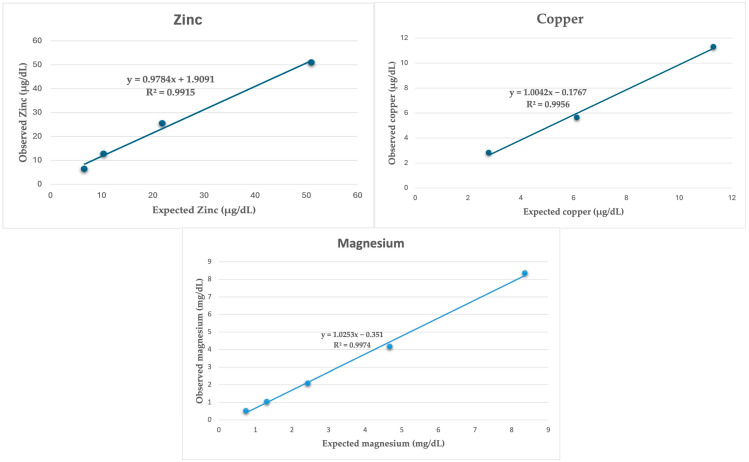
Linearity under the dilution of a horse saliva sample for zinc, copper, and magnesium.

When saliva samples were diluted serially with ultrapure water, they showed high linearity in the regression, with correlation coefficients close to 1 for Zn, Cu, and Mg measurements (Figure 1). The recovery study in the case of Fe showed recovery percentages ranging from 90.74 to 109.06%. The LOD for Cu, Mg, and Fe was set at 0.86 µg/dL, 0.38 µg/dL, and 13 µg/dL. The LOD of the Zn assay could not be calculated since all measurements with ultrapure water gave negative values.

Correlation with the gold-standard ICP-MS indicated a high positive correlation between determinations of the automated assays for Cu (r = 0.92, *p* < 0.0001) and Mg (r = 0.93, *p* < 0.0001), while Zn (r = 0.70, *p* = 0.004) and Fe (r = 0.72, *p* = 0.004) showed a moderate positive correlation.

### 3.3. Changes in Salivary Concentrations of Zinc, Copper, Magnesium, and Iron in Horses with Equine Gastric Ulcer Syndrome

**Figure 2:**
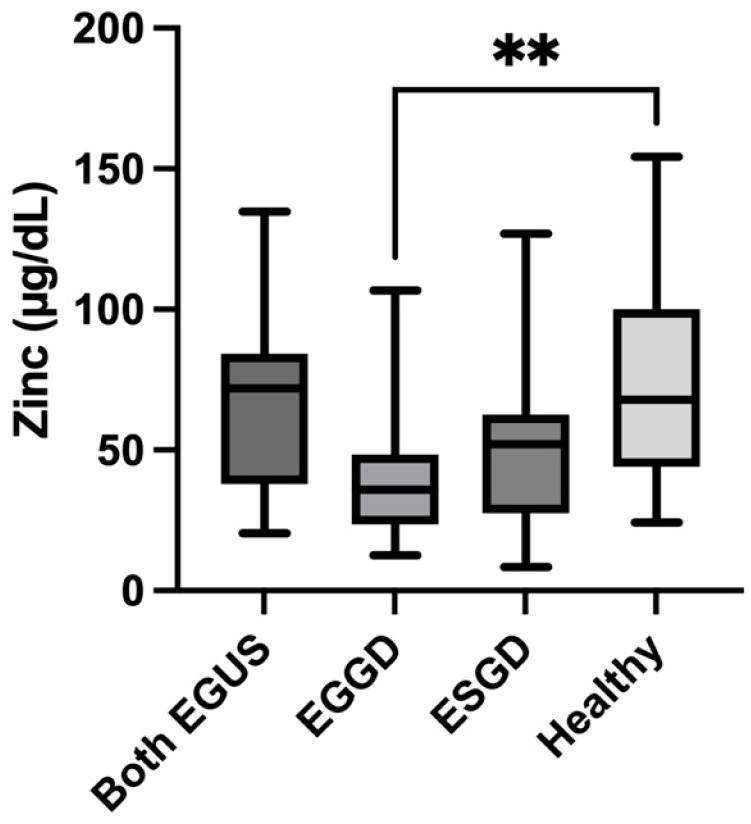
Values of concentrations of Zinc in the saliva of horses with both Equine Glandular Gastric Disease (EGGD) and Equine Glandular Gastric Disease (ESGD), horses with only EGGD, horses with only ESGD, and healthy horses. Horizontal lines represent the median concentrations, boxes indicate the 10–90 percentiles, and whiskers represent the ranges. ** *p* < 0.01.

Horses diagnosed solely with EGGD presented significantly lower levels of Zn (median = 52.15 µg/dL, IQR = 27.53–62.6) compared with healthy horses (median = 67.8, IQR = 44.1–100) (*p* = 0.005) and there was a tendency of Zn to decrease in horses with ESGD (Figure 2).

**Figure 3:**
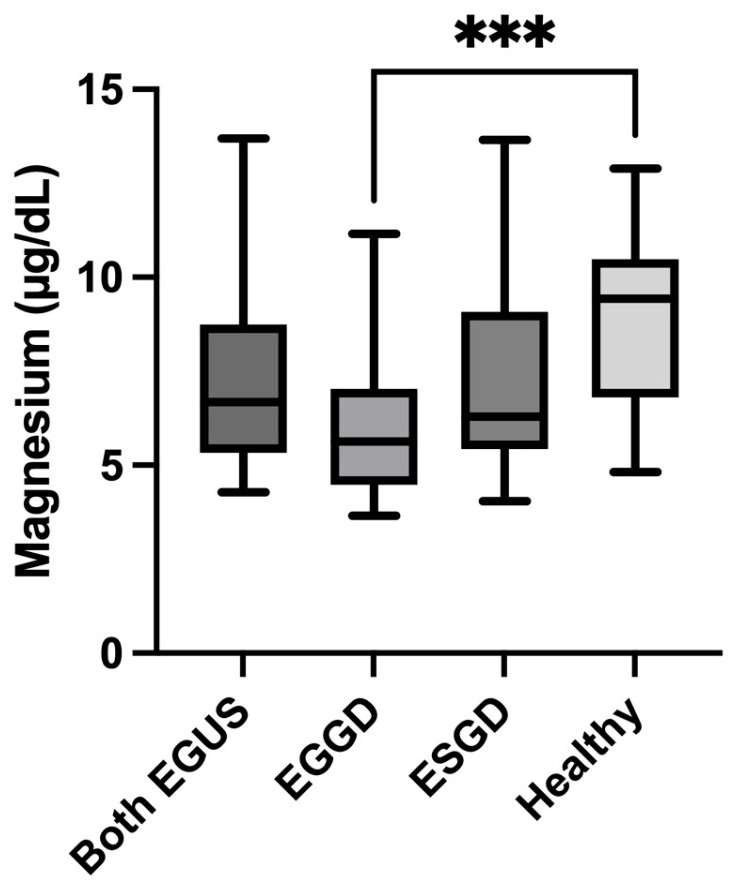
Values of concentrations of Magnesium in the saliva of horses with both Equine Glandular Gastric Disease (EGGD) and Equine Glandular Gastric Disease (ESGD), horses with only EGGD, horses with only ESGD, and healthy horses. Horizontal lines represent the median concentrations, boxes indicate the 10–90 percentiles, and whiskers represent the ranges. *** *p* < 0.05.

A similar tendency was observed for Mg, which was significantly reduced in horses with EGGD (median = 5.63 µg/dL, IQR = 4.48–7.02) compared with healthy horses (median = 9.43 µg/dL, IQR = 6.81–10.48) (*p* = 0.0008) (Figure 3).

**Figure 4:**
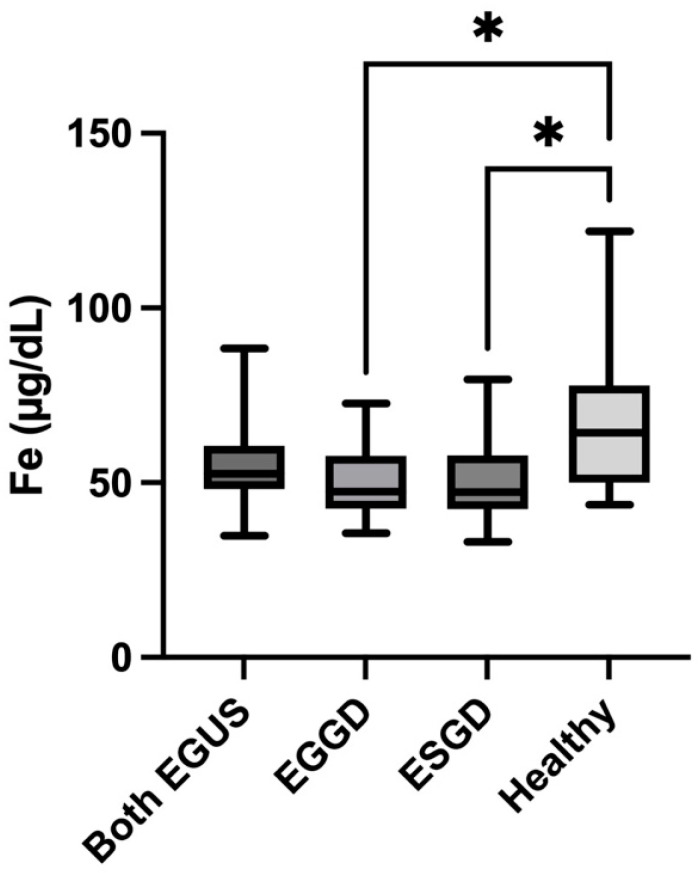
Values of concentrations of Iron in the saliva of horses with both Equine Glandular Gastric Disease (EGGD) and Equine Glandular Gastric Disease (ESGD), horses with only EGGD, horses with only ESGD, and healthy horses. Horizontal lines represent the median concentrations, boxes indicate the 10–90 percentiles, and whiskers represent the ranges. * *p* < 0.05.

The concentrations of Fe were significantly lower in horses with ESGD (median = 47.4 µg/dL, IQR = 42.5–57.7) (*p* = 0.03) and EGGD (median = 47.3 µg/dL, IQR = 42.4–57.8) (*p* = 0.01) compared with healthy horses (median = 64.3 µg/dL, IQR = 50–77.8) (Figure 4).

**Figure 5:**
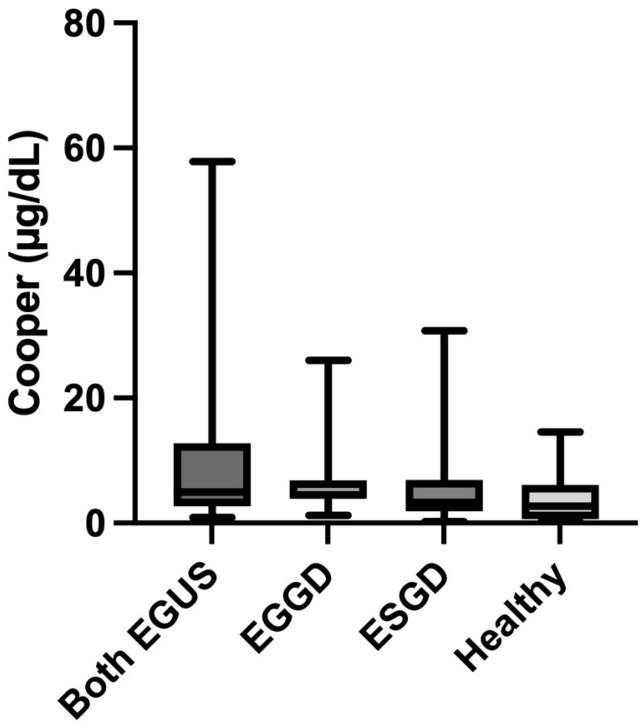
Values of concentrations of Cooper in the saliva of horses with both Equine Glandular Gastric Disease (EGGD) and Equine Glandular Gastric Disease (ESGD), horses with only EGGD, horses with only ESGD, and healthy horses. Horizontal lines represent the median concentrations, boxes indicate the 10–90 percentiles, and whiskers represent the ranges.

No significant variations were observed in the measurement of salivary Cu in any of the groups, although horses with both types of EGUS had increased concentrations of this analyte (Figure 5).

## 4. Discussion

This study used spectrophotometric assays for the measurements of Zn, Cu, Mg, and Fe in the saliva of horses and detected changes in the concentration of these analytes in horses with EGUS. The assays used in this report have two main advantages. One is the fact that they do not need any pre-processing action or treatment for the samples. The second one is that they are automated, although they can also be adapted to other formats, such as 96-well plates or manual spectrophotometers. The no need for sample treatment, and the possibility of automation allow the processing and analysis of a large number of samples in a short time, making these assays suitable for routine measurements of these analytes.

The assays used in this study showed CVs lower than the limit recommended for automated methods [31] and, therefore, were precise. In addition, when accuracy testing was performed, they were linear after serial dilutions and, in the case of Fe, showed high spiking recovery rates. Also, the automated methods of our report showed a positive linear relationship with ICP-MS, which is considered a gold-standard method for the measurement of these analytes, although it needs previous digestion and acidification or deproteinization of the sample. These results were similar to those found when the same assays were validated for the measurement of Zn and Cu in the saliva of pigs [32]. In addition, our results agree with previous studies in which these analytes were measured in different types of human specimens, such as saliva, serum, or semen, without any previous sample processing [33,34].

In our study, horses diagnosed with EGUS showed lower concentrations of Zn in saliva compared with healthy horses, although these differences were only significant in the case of the glandular gastric form. Low values of Zn, probably due to a reduced uptake, have been found to be associated with gastric ulcers in rats [11]. On the other hand, Zn administration has been used for the treatment of gastric ulcers in humans and rats [11], and increasing Zn levels using oral Zn sulfate has been found to reduce ulcer size and increase ulcer healing rates [7,9]. As Zn is important in ulcer healing and as reduced Zn absorption has been found during ulcer treatment with omeprazole [35], further research into Zinc supplementation during treatment should be conducted.

This study also found a significantly lower concentration of Mg in EGGD-diagnosed horses compared to that in healthy control horses. Magnesium has been found to be ulcer-protective on the stomach by reducing the number of gastric parietal cells and increasing the function of the gastric mucous cells [13]. It is widely used in humans for its antacid effect, decreasing acid production [36].

In addition to the relation between lower concentrations of Zn and Mg and the presence of increased acid production that can lead to gastric ulcers, there could also be a link between these trace elements and the immune system’s function. In this line, the lower values that presented both trace elements in horses with EGGD, in which a possible impairment of the immune system and inflammation has been indicated, could be related to the involvement of Zn and Mg in the regulation of immune function and inflammation. For example, Zn can decrease the generation of inflammatory mediators and cytokines [37], and deficiency in Mg can lead to temporary or long-term immune dysfunction [38].

Concentrations of Cu did not show significant changes in horses with EGUS compared to healthy horses, although the mean values in EGUS were higher. Higher values of Cu have been found in the serum of humans with peptic ulcer disease compared to healthy controls, probably in order to produce protection against the disease. Cu has a protective effect by the inhibition of prostaglandin and thromboxanes, which are mediators of inflammation, and it further can scavenge superoxide radicals and, therefore, protect from oxidative damage [14]. A similar tendency was observed in our study since EGUS horses had higher levels of Cu than controls. Therefore, further investigation using a large cohort of horses with the disease should be performed to confirm this tendency and also elucidate if Cu has different dynamics in the different types of EGUS.

In this study, significantly lower saliva Fe levels were found in horses suffering from EGGD and ESGD. The low concentrations could be due to a decreased absorption of Fe, which has been described in humans suffering from gastric ulcers [39]. Most studies on Fe and gastric ulcers in humans describe ulceration due to oral supplementation of iron [20,40,41], which are not described in horses. In addition, it could be of interest to evaluate if this reduction of Fe could be related to the impairment in immune function and development of inflammation that has been reported in EGGD since Fe is related to immune regulation [42].

Overall, these results indicate changes in saliva trace elements in horses diagnosed with EGUS. These changes, especially in EGGD, could reflect the physiopathological mechanisms involved in this process. However, it is important to point out that this is a pilot study, and these results should be confirmed in a larger population of horses. In addition, further studies should be undertaken to evaluate these trace elements in the saliva of horses before and after ulcer treatment, as ulcer medication in humans points towards a reduced uptake of trace elements with treatment [9,11]. Further, it would be of interest to explore if supplementation of these trace elements can improve ulcer healing in horses with EGUS and also if their measurements in saliva could be of use as biomarkers of this disease, for example, for treatment monitoring. These studies would help to elucidate and clarify the possible practical applications of measuring trace elements in saliva in horses with EGUS. Additionally, future studies should aim to elucidate whether these variations are indeed a direct result of the ulcers and/or if they contribute to their development. Such investigations will be of high interest in understanding the physiopathological mechanisms involved in EGUS and exploring the potential diagnostic and therapeutic applications of monitoring trace elements in saliva.

## 5. Conclusions

In conclusion, the assays evaluated in this study were able to measure Zn, Cu, Mg, and Fe in the saliva of horses in a simple, fast, and precise manner. In addition, these assays were able to detect changes in these trace elements in horse saliva between horses diagnosed with EGUS and healthy control horses. These changes were a decrease in Zn and Mg levels in saliva from horses diagnosed with EGGD compared to healthy control horses and a decrease in Fe in horses diagnosed with both ESGD and EGGD compared to control horses. Further studies should be performed to clarify the possible practical applications of these trace elements in saliva as biomarkers and their supplementation’s effect during the treatment of this highly prevalent disease.

## Data Availability

The data presented in this study are available on request from the corresponding author.
